# An Integrative Omics Approach Reveals Involvement of *BRCA1* in Hepatic Metastatic Progression of Colorectal Cancer

**DOI:** 10.3390/cancers12092380

**Published:** 2020-08-22

**Authors:** Daniela Gerovska, Gorka Larrinaga, Jon Danel Solano-Iturri, Joana Márquez, Patricia García Gallastegi, Abdel-Majid Khatib, Gereon Poschmann, Kai Stühler, María Armesto, Charles H. Lawrie, Iker Badiola, Marcos J. Araúzo-Bravo

**Affiliations:** 1 Computational Biology and Systems Biomedicine Group, Biodonostia Health Research Institute, Calle Doctor Beguiristain s/n, 20014 San Sebastián, Spain; daniela.gerovska@biodonostia.org; 2 Computational Biomedicine Data Analysis Platform, Biodonostia Health Research Institute, Calle Doctor Beguiristain s/n, 20014 San Sebastián, Spain; 3 Department of Nursing I, Faculty of Medicine and Nursing, University of the Basque Country (UPV/EHU), 48940 Leioa, Bizkaia, Spain; gorka.larrinaga@ehu.eus; 4 Department of Physiology, Faculty of Medicine and Nursing, University of the Basque Country (UPV/EHU), 48940 Leioa, Bizkaia, Spain; 5 BioCruces Health Research Institute, 48903 Barakaldo, Bizkaia, Spain; jondanel.solanoiturri@osakidetza.eus; 6 Department of Anatomic Pathology, Cruces University Hospital, University of the Basque Country (UPV/EHU), 48903 Barakaldo, Bizkaia, Spain; 7 Department of Cell Biology and Histology, Faculty of Medicine and Nursing, University of Basque Country (UPV/EHU), 48940 Leioa, Spain; joana.marquez@ehu.eus (J.M.); patricia.garcia@ehu.eus (P.G.G.); 8 University of Bordeaux, Allée Geoffroy St Hilaire, 33615 Pessac, France; INSERM, LAMC, UMR 1029, Allée Geoffroy St Hilaire, 33615 Pessac, France; majid.khatib@inserm.fr; 9 Institute of Molecular Medicine, Proteome Research, Medical Faculty, Heinrich-Heine-University, 40225 Düsseldorf, Germany; gereon.poschmann@hhu.de (G.P.); kai.stuehler@hhu.de (K.S.); 10 Molecular Proteomics Laboratory, Biologisch-Medizinisches Forschungszentrum, Heinrich-Heine-University, 40225 Düsseldorf, Germany; 11 Molecular Oncology Group, Biodonostia Health Research Institute, 20014 San Sebastián, Spain; maria.armesto@biodonostia.org (M.A.); charles.lawrie@biodonostia.org (C.H.L.); 12 Radcliffe Department of Medicine, University of Oxford, Oxford OX3 9DU, UK; 13 IKERBASQUE, Basque Foundation for Science, Calle María Díaz Harokoa 3, 48013 Bilbao, Spain; 14 CIBER of Frailty and Healthy Aging (CIBERfes), 28029 Madrid, Spain; 15 Computational Biology and Bioinformatics Group, Max Planck Institute for Molecular Biomedicine, Röntgenstr. 20, 48149 Münster, Germany

**Keywords:** colorectal cancer, liver metastasis, tumor microenvironment, tissue microarray, transcriptomics, miRNAs, proteomics, BRCA1, BRCA1-associated genome surveillance complex

## Abstract

(1) Background & Aims: The roles of different cells in the tumor microenvironment (TME) are critical to the metastatic process. The phenotypic transformation of the liver cells is one of the most important stages of the hepatic metastasis progression of colorectal cancer (CRC). Our aim was to identify the major molecules (i.e., genes, miRNAs and proteins) involved in this process. (2) Methods: We isolated and performed whole-genome analysis of gene, miRNA, and protein expression in three types of liver cells (Ito cells, Kupffer cells, and liver sinusoidal endothelial cells) from the TME of a murine model of CRC liver metastasis. We selected the statistically significant differentially expressed molecules using the Student’s t-test with Benjamini-Hochberg correction and performed functional statistically-significant enrichment analysis of differentially expressed molecules with hypergeometric distribution using the curated collection of molecular signatures, MSigDB. To build a gene-miRNA-protein network centered in Brca1, we developed a software package (miRDiana) that collects miRNA targets from the union of the TargetScan, MicroCosm, mirTarBase, and miRWalk databases. This was used to search for miRNAs targeting *Brca1*. We validated the most relevant miRNAs with real-time quantitative PCR. To investigate BRCA1 protein expression, we built tissue microarrays (TMAs) from hepatic metastases of 34 CRC patients. (3) Results: Using integrated omics analyses, we observed that the *Brca1* gene is among the twenty transcripts simultaneously up-regulated in all three types of TME liver cells during metastasis. Further analysis revealed that *Brca1* is the last BRCA1-associated genome surveillance complex (BASC) gene activated in the TME. We confirmed this finding in human reanalyzing transcriptomics datasets from 184 patients from non-tumor colorectal tissue, primary colorectal tumor and colorectal liver metastasis of the GEO database. We found that the most probable sequence of cell activation during metastasis is Endothelial→Ito→Kupffer. Immunohistochemical analysis of human liver metastases showed the BRCA1 protein was co-localized in Ito, Kupffer, and endothelial cells in 81.8% of early or synchronous metastases. However, in the greater part of the metachronous liver metastases, this protein was not expressed in any of these TME cells. (4) Conclusions: These results suggest a possible role of the co-expression of BRCA1 in Ito, Kupffer, and sinusoidal endothelial cells in the early occurrence of CRC liver metastases, and point to BRCA1 as a potential TME biomarker.

## 1. Introduction

Colorectal cancer (CRC) is the third most common cancer amongst men in developed countries and the second most common amongst women [1]. Most CRCs occur on an existing polyp in the lining of the colon or rectum, which evolves into a malignant tumor. Malignant tumor cells enter the bloodstream and metastasize preferentially in the liver, which accounts for over 70% of metastatic CRC cases. The walls of hepatic sinusoids are lined by three cell types: liver sinusoidal endothelial cells (Es), known as LSECs, Kupffer cells (Ks), and hepatic stellate cells, formerly known as fat-storing, perisinusoidal, vitamin A-rich cells, lipocytes or Ito cells (Is).

Until recently, the treatment of liver metastases focused on fighting the tumor cells that had acquired metastatic capabilities, but this trend has been altered to some extent thanks to the findings related to the tumor microenvironment (TME) rationale [2], based on the “seed and soil” theory [3] whereby tumor cells act upon the cells of the receiving organ, which in turn modulate their phenotype to produce an environment conducive to tumor progression. During tumor development, cancer cells secrete molecular signals to the environment and change the phenotype of the surrounding cells, creating new supporting tumor-survival and growth conditions [4].

It is well known in CRC that endothelial cells [5] and liver stellate cells [6,7] actively facilitate metastatic tumor cells to colonize and propagate in the liver. However, this phenomenon is not unidirectional: once activated, liver endothelial cells act upon the tumor cells to help transform them into phenotypes with stronger invasive and colonizing ability [5], creating a feedback loop containing a number of different cells.

**Figure 1:**
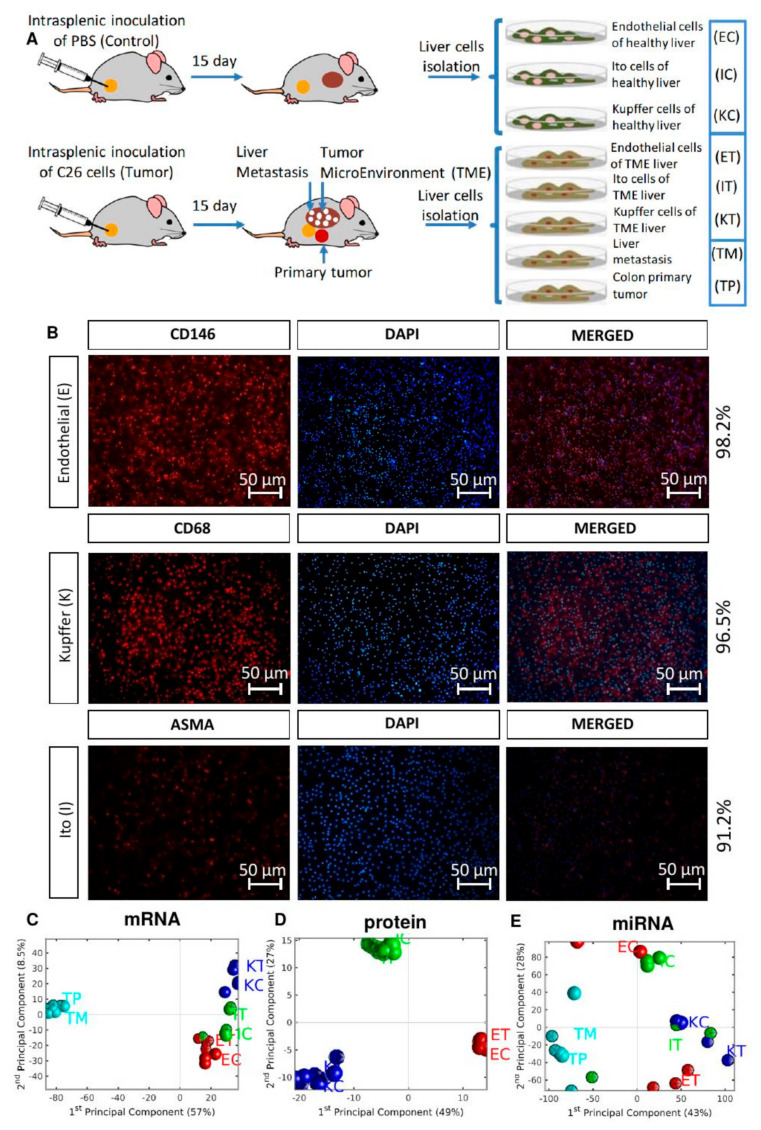
Experimental design and global omics analysis. (**A**) Two groups of mice were inoculated with C26 murine colon cancer cells and with PBS (Control). After 15 days, the mice were perfused, and after PercollV R gradient centrifugation, three types of liver cells were collected, namely liver sinusoidal endothelial cells (E), Ito cells (I) and Kupffer cells (K), from control (C) and TME (T), and isolated to perform omics experiments (gene expression, miRNA expression microarrays and proteomics). TP and TM denote the CRC primary and tumor liver metastasis cells, respectively. (**B**) Cell purity was checked via immunochemistry using specific antibodies to detect endothelial (CD146), Kupffer (CD68), and Ito (ASMA) cells. (**C**–**E**) PCA plots of mRNA, protein and miRNA expression. Red, blue, and green symbols mark endothelial, Kupffer, and Ito cells, respectively. TME cells are depicted with dodecahedra, and controls with spheres. For each mRNA sample we used 4 biological replicates; for each miRNA sample 3 biological replicates, except 2 for ECs and KTs; for the proteomics data we used 9 biological replicates for ECs, 7 for ETs, 6 for ICs and ITs, and 5 for KCs and KTs.

To understand the liver TME network, we created a murine model of CRC liver metastasis and performed an integrative omics approach encompassing RNA and microRNA (miRNA) transcriptomics and proteomics data from Ito (I), Kupffer (K), and sinusoidal endothelial (E) cell populations of healthy control mice (C) and induced CRC, from which we collected the TME (T), the CRC primary (TP), and the tumor liver metastasis (TM) cells Figure 1A. We validated our findings at the protein level by constructing Tissue Microarrays (TMAs) of human 34 liver metastasis samples from patients with CRC.

## 2. Materials and Methods

### 2.1. Animals

Balb/c mice (6- to 8-week-old males) were obtained from Charles River Laboratories Spain SA (Barcelona, Spain). All procedures were approved by the Ethical Committee for Animal Experimentation of the University of the Basque Country (EHU/UPV) under the code CEEA/391/2015/BADIOLA ETXABURU in accordance with institutional, national and international guidelines regarding the protection and care of animal use for scientific purposes. Mice were kept in the animal facility of EHU/UPV and had access to standard chow and water ad libitum.

### 2.2. Colorectal Cancer Cells

Murine CRC C26 cells (ATCC, Manassas, VA, USA) syngeneic with Balb/c mice were grown under standard conditions in RPMI medium (Sigma-Aldrich; St. Louis, MO, USA) supplemented with 10% fetal bovine serum (FBS), penicillin (100 U/mL), streptomycin (100 mg/mL) and amphotericin B (0.25 mg/mL), all purchased from Life Technologies, Carlsbad, CA, USA.

### 2.3. Control and Tumor-Activated Hepatic Cell Isolation and Culture

Control and tumor-activated primary cultures of hepatic cells, LSECs, Ito cells and Kupffer cells were isolated from livers with CRC metastasis or from healthy controls. Balb/c mice were anesthetized with isofluorano and underwent surgical incisions on their left broadside. Mice were inoculated into the spleen at the incision sites using 2 × 10^6^ of C26 colon carcinoma cells. Control mice were inoculated with PBS. Fourteen days later, all mice were sacrificed, and the liver cells were removed and purified by differential centrifugation. In brief, the mice were perfused with Clostridium histolyticum collagenase P (Sigma-Aldrich; St. Louis, MO, USA) through the cava vein, and the obtained cell suspension was twice centrifuged, resulting in a parenchymal (PC)-enriched pellet and a non-PC-enriched supernatant. The non-PC-enriched supernatant was layered on Percoll gradients (25% on top of 50% *w/v*) to obtain LSECs and on Percoll gradients (33% on top of 50% *w/v*) to obtain Ito cells. After centrifugation, the interphase between the two density cushions was collected and contained purified non-PC enriched LSECs in the first gradient and Ito cells in the second one. Both solutions contained Kupffer cells, which were further separated by adherence assays. All cell fractions were washed and cultured with RPMI 1640 medium (Sigma-Aldrich; St. Louis, MO, USA) supplemented with 10% FBS and used in different experiments a maximum of 24 h after isolation (Life technologies, Carlsbad, CA, USA).

### 2.4. Omics Analysis

One-hundred ng of total RNA from each fraction was labeled and hybridized onto Agilent mouse RNA and miRNA microarrays, Release 18.0 (Agilent Technologies, Santa Clara, CA, USA) following the standard Agilent Protocol with RNA and miRNA Complete Labelling and Hybridization Kits, including Agilent RNA Spike-ins. Results were scanned using an Agilent Microarray Scanner G2565CA. Scanned TIFF image files were processed using Agilent Feature Extraction Software (v10.7.3.1) to extract the raw data. Proteomics analysis was performed via mass spectrometry as described previously [8]. Briefly, samples were shortly separated in a polyacrylamide gel, and proteins were reduced with dithiothreitol, alkylated with iodoacetamide, and digested with trypsin. After extraction, the peptides were finally resuspended in 0.1% trifluoroacetic acid. Approximately 500 ng of peptides were subsequently separated on an Ultimate 3000 rapid separation liquid chromatography system (Thermo Fisher Scientific, Dreieich, Germany) and analyzed on an Orbitrap Elite (Thermo Fisher Scientific, Dreieich, Germany) hybrid mass spectrometer. All omics data were normalized with the quantile method. PCA and the hierarchical clustering of genes and samples were performed with one minus correlation metric and the unweighted average distance linkage method. To find the statistically significant molecules and their GO enrichment, we used the method described previously [9] with the selection threshold θ*_DEG_*= θ*_DEM_*= 4, θ*_DEP_*= 2, and significance threshold α*_DEG_*= α*_DEM_*= α*_DEP_*= 0.001. For each mRNA sample, we used 4 biological replicates; for each miRNA sample 3 biological replicates, except 2 for ECs and KTs; for the proteomics data 9 biological replicates for ECs, 7 for ETs, 6 for ICs and ITs, and 5 for KCs and KTs.

### 2.5. miRNA Validation with miRNA RT-qPCR

The microarray data of three of the most relevant miRNAs were validated with real-time PCR (RT-qPCR) using a miRCURY LNATM Universal RT miRNA PCR system (Exiqon, Denmark), following the manufacturer’s instructions. Also, 50 ng of total RNA was used for cDNA synthesis using a Universal cDNA synthesis kit II (Exiqon, Denmark). cDNA was amplified in triplicate in a 7900 HT Fast Real Time System (Life Technologies, Carlsbad, CA, USA) using miRNA LNA primer sets for the specified miRNAs (Exiqon, Denmark) and ExiLENT SYBR^®^ Green master mix (Exiqon, Denmark). The expression of miRNAs was normalized to the *SNORD68* control gene, and the relative expression was calculated with the 2^−ΔΔCt^ method.

### 2.6. Protein-Gene Correlation Analysis

For each gene of the transcriptomics dataset, we selected the probe with the highest expression variance across all samples. We selected the gene and protein with the same official names. We used a robust regression technique [10] to estimate the fit of protein vs. gene expression.

### 2.7. Algorithm to Search for miRNAs Targeting Genes

To search for miRNA target genes, we developed software in MATLAB^®^ (MathWorks^®^), miRDiana that collects the union of mouse validated targets from the TargetScan [11], MicroCosm [12], mirTarBase [13] and miRWalk 2.0 [14] databases. Firstly, the software downloads each database and preprocesses by standardizing the miRNA and gene names. It strips the miRNA names from the species ids and converts the gene names to the official symbols of the National Center for Biotechnology Information (NCBI) database. Next, for each potential gene target, it calculates an incidence matrix with all the miRNAs of each database targeting such genes. Finally, it builds a consensus matrix with the cases of the appearance of each miRNA in the four analyzed miRNA databases.

### 2.8. Gene-miRNA-Protein Network Centered in Brca1

We applied our software to search for miRNAs targeting genes to search for miRNAs targeting *Brca1*. Gene-miRNA-protein networks were built for each control-TME sample using the gene expression of *Brca1* surrounded by three concentric rings to depict the expression miRNAs that target this gene, and in turn surrounded by the protein and gene expression of the genes targeted by these miRNAs. To reduce the number of genes in the outermost double ring, we selected genes with a difference of expression between ET and EC less than 0.3 on a log_2_ scale.

### 2.9. CRC Patients and Samples

All experiments in this study comply with the current Spanish and European Union legal regulations. Samples and data from patients were provided by the Basque Biobank for Research-OEHUN. All patients were informed and gave written consent for the use of their tissue in this project by signing a document approved by the Ethical and Scientific Committees of the Basque Country Public Health System (CEIC 11/51 and CEIC 18/37).

To build TMAs, paraffin-embedded liver metastases from 34 CRC patients were identified and collected from 28 males (mean age: 65.1 years) and 6 females (mean age: 64.7 years). Eleven of these samples presented with synchronous metastasis (i.e., they were detected in the moment of the first diagnosis (Stage IV)) and the remaining 23 cases had metachronous metastases with four patients diagnosed with Stage I, ten with Stage II, and nine with Stage III at the time of clinical presentation.

The TMAs were constructed using a Tissue Microarrayer Model MTA1 system using two 1.5 mm cores selected from areas of the tumor with high tumor/stroma ratios and avoiding necrotic islands. Immunohistochemical data were analyzed with SPSS 23.0 using χ^2^ test to assess the statistical significance of the differences of BRCA1 expression between different liver metastatic samples.

### 2.10. BRCA1 Immunostaining of TMA

TMAs were incubated during 20 min at 60 °C with antigen retrieval solution (Sigma, St. Louis, MO, USA), fixed in formaldehyde, blocked with goat serum, and incubated at 4 °C for 8 h with different antibodies to detect each cell type: LSECs with mouse anti-CD146 antibody (ab24577, Cambridge, UK), Ito cells with mouse anti-alpha smooth muscle actin antibody (ab7817, Abcam, Cambridge, UK), and Kupffer cells with mouse anti-CD68 antibody (ab31630, Abcam, Cambridge, UK). BRCA1 was detected with rabbit anti-BRCA1 antibody (orb48292, Biorbyt, Cambridge, UK). All antibodies were diluted at 1:100. Then, the samples were washed with PBS-0.3% Tween and incubated with the secondary antibody diluted 1:1000 for one minute; goat anti-rabbit Alexa 433 (Sigma, St. Louis, MO, USA) to detect BRCA1 and goat anti-mouse Alexa 594 to detect each cell type. After washing, the samples were incubated with DAPI diluted 1:1000 for 10 min.

## 3. Results

### 3.1. The Global Differences between Control and TME Samples are Most Pronounced at miRNA Expression Level

Immunochemistry was used to confirm the purity of specific cells on our cell fractions using CD146, CD68, and ASMA to detect endothelial, Kupffer, and Ito cells, respectively. We found that all of the isolated cell fractions had purity values higher than 90% Figure 1B. At mRNA Figure 1C and protein Figure 1D levels, the three different TME cell types were observed to cluster together, irrespective of whether cells were tumor-associated or not. At the miRNA level, however, the variability was higher, with more pronounced discrimination between TME and control samples, with all TME samples displaying positive coordinates in the first Principal Component Figure 1E. We used pairwise scatter plots to analyze differences between the TME and control samples at the three molecular levels. We observed that TME and control samples displayed very similar patterns at both gene expression Figure S1A–G and protein Figure S1C–I levels, again showing more pronounced differences at the miRNA level Figure S1B–H. The proteomics data reveals that Kupffer cells have less significant DEPs between control and TME samples than the other liver cells, the top down- regulated protein in endothelial TME cells is carbamoyl-phosphate synthetase 1, Cps1, localized in the inner mitochondrial matrix (Figure S2A). Among the up-regulated is the superoxide dismutase 1 Sod1 (Figure S2B), described to promote the epithelial-mesenchymal transition (EMT) of pancreatic cancer cells via activation of the H2O2/ERK/NF-κB axis [15] and to be involved in aging [16]. The TME versus control dysregulated proteins are not shared between the different liver cells types (Figure S2). The correlation analysis between protein and gene expression reveals a slight positive correlation between the two molecular measurements (Figure S3). The regression of the protein expression versus the gene expression of the merged data of all the samples has a slope of 0.11 and a Pearson’s correlation coefficient of 0.23. The projection of the protein-gene expression points over the orthogonal regression line shows a bias due the enrichment of lower expressed proteins in the region of middle expressed genes. Such behavior is independent of the cell type (Figure S3A–M). Thus, the proteomics data are slightly positively correlated with the transcriptomics data. The TME does not appear to have a strong molecular fingerprint at the global profile level. Consequently, we searched for individual tumorogenesis-related molecules by analyzing differentially expressed genes (DEGs), differentially expressed miRNAs (DEMs), and differentially expressed proteins (DEPs) between TME and control samples in the three liver cell types.

### 3.2. miR-21a, miR-146a, miR-16 and miR-29a Are among the miRNAs Simultaneously Down-Regulated in All the TME Cells

**Figure 2:**
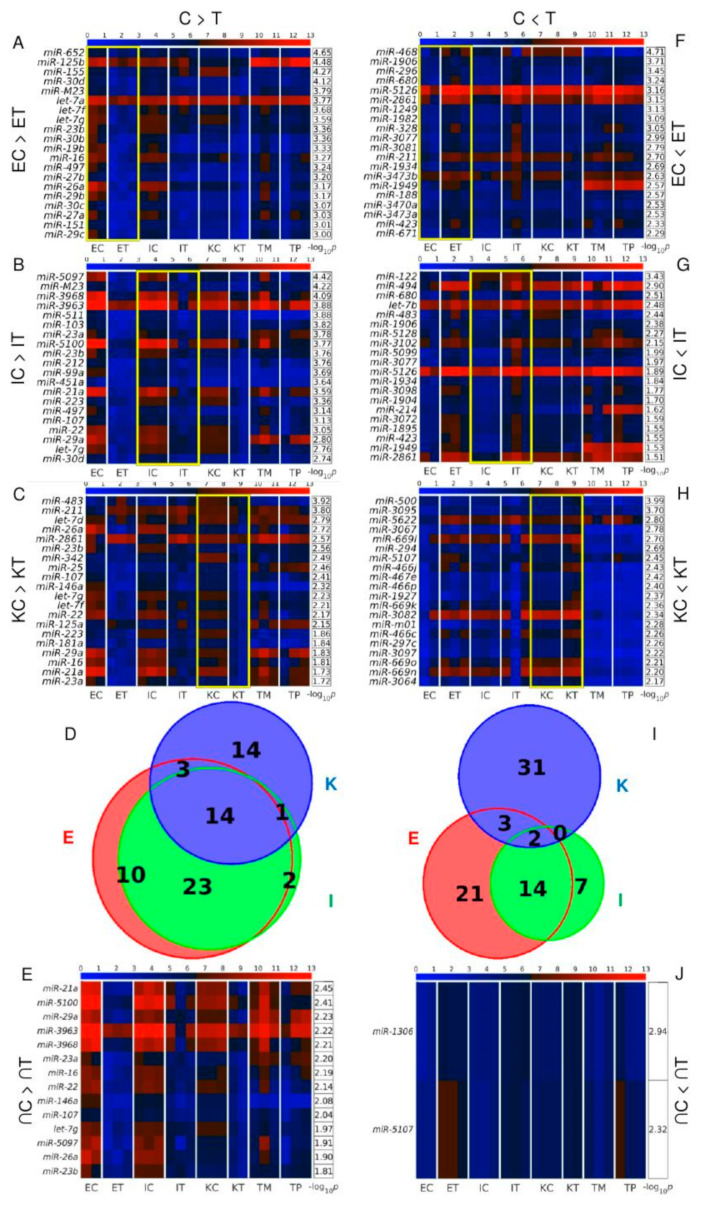
Differentially expressed miRNAs (DEMs) between control and TME cells. Heatmaps of the expression of the 20 top-ranked in decreasing order of significance, TME DEMs: (**A**–**C**) down-regulated, and (**F**–**H**) up-regulated, in endothelial, Ito, and Kupffer cells. Color bars codify miRNA expression on a log_2_ scale. Higher miRNA expression corresponds to a redder color. Yellow frames mark the samples used to find the DEMs. The –log_10_ (*p*-value) of the DEMs are presented in tables to the right of each heatmap. Euler-Venn diagrams of the (**D**) down-regulated and (**I**) up-regulated TME DEMs shared by the endothelial, Ito, and Kupffer cells are shown. Heatmaps of the expressions of the (**E**) fourteen miRNAs down-regulated and (**J**) two miRNAs up-regulated in the three types of TME cells are also shown. The –log_10_ of the average of the *p*-values across the three cell type comparisons are presented in tables to the right of each heatmap. In all subplots, the samples are denoted with E, I, and K for endothelial, Ito, and Kupffer cells, C and T, for control and TME cells, and TP and TM denote the CRC primary and tumor liver metastasis cells, respectively. For each miRNA sample, we used 3 biological replicates except 2 for ECs and KTs.

We identified DEMs between control and TME cells Figure 2A–H, including 14 commonly down-regulated in TME cells Figure 2D,E, and 2 commonly up-regulated (*miR-1306*, *miR-5107*) DEMs in TME cells Figure 2I,J. Interestingly, *miR21a*, *miR-23a*, *miR-23b*, *miR-29a*, and *miR-3963* which were down-regulated had a strong signal in both CRC primary tumors and tumor liver metastasis.

### 3.3. Brca1 and Sp110 Genes Are among the Twenty Transcripts Simultaneously Up-Regulated in All the TME Cells

**Figure 3:**
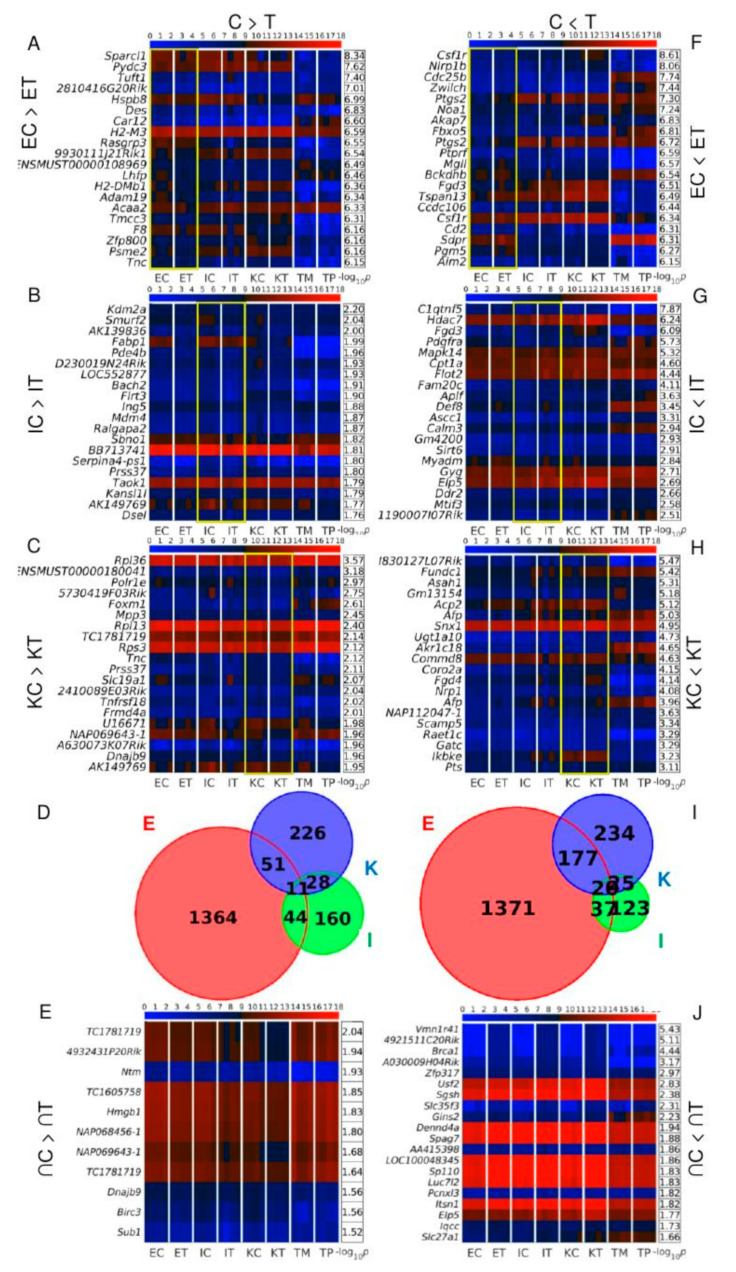
Differentially expressed genes (DEGs) between control and TME cells. Heatmaps of the expression of the 20 top-ranked, in decreasing order of significance, TME DEGs: (**A**–**C**) down-regulated, and (**F**–**H**) up-regulated, in endothelial, Ito, and Kupffer cells. Color bars codify the gene expression on a log_2_ scale. Higher gene expression corresponds to a redder color. Yellow frames mark the samples used to find the DEGs. The –log_10_ (*p*-value) of the DEGs are presented in tables to the right of each heatmap. Euler-Venn diagrams of the (**D**) down-regulated and (**I**) up-regulated TME DEMs shared by the endothelial, Ito, and Kupffer cells are shown. Heatmaps of the expressions of the (**E**) eleven genes down-regulated and (**J**) twenty genes up-regulated in the three types of TME cells are also shown. The –log_10_ of the average of the *p*-values across the three cell type comparisons are presented in tables to the right of each heatmap. In all subplots, the samples are denoted with E, I, and K for endothelial, Ito, and Kupffer cells, C and T for the control and TME cells, and TP and TM denote the CRC primary and tumor liver metastasis cells, respectively. For each mRNA sample, we used 4 biological replicates.

Analysis of DEGs between control and TME cells Figure 3A–H revealed that endothelial cells had higher levels of DEGs than the other cells types with 1364 transcripts down- and 1371 up-regulated in the TME cells of Figure 3D,I. There were 11 DEGs that were commonly down-regulated in all TME cells of Figure 3D,E and 20 transcripts commonly up-regulated in Figure 3I,J. Among the up-regulated TME transcripts of Figure 3J, the most relevant are the breast cancer 1 gene *Brca1*, and *Sp110*. *Sp110* is a member of the SP100/SP140 family of nuclear body proteins that encodes a leukocyte-specific nuclear body component regulating genes involved in the inflammatory response and miRNA expression in macrophages. *Sp110* has been reported to have immune and apoptosis-related miRNAs such as *miR-125a*, *miR-146a*, *miR-155*, *miR-21a* and *miR-*99b [17]. Interestingly, we also observed the simultaneous down-regulation of *miR-146a* and *miR-21a* in TME in the three types of studied liver cells of Figure 2E.

### 3.4. Slurp1 and Acnat2 Are Highly Positively, and Tpt1, Emd and Rnf167 Are the Top Genes Highly Negatively Correlated with Brca1 across All Samples

**Figure 4:**
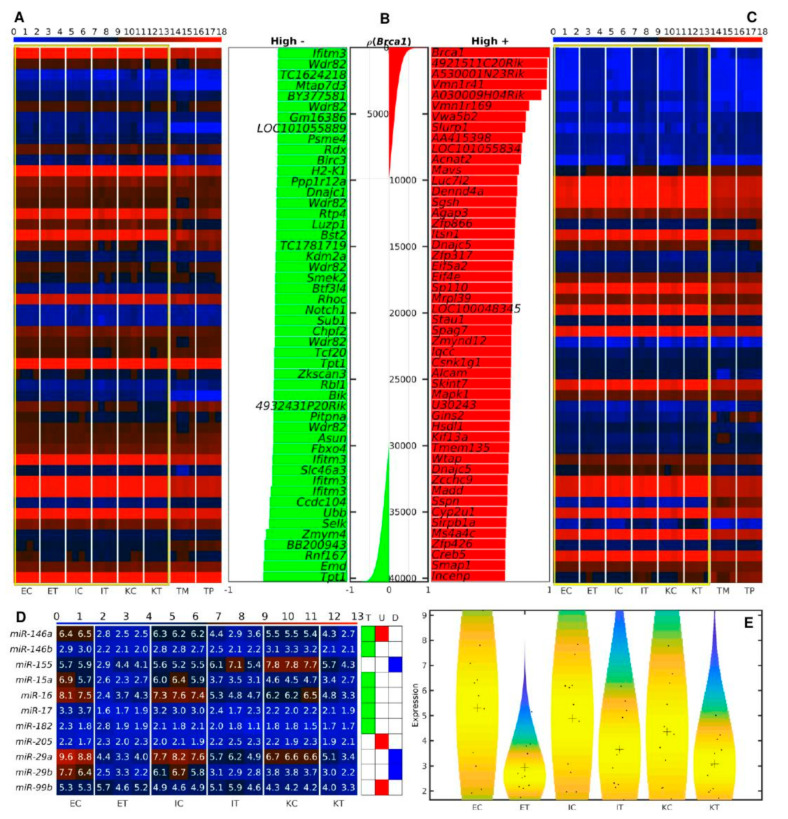
Gene expression correlation with *Brca1*. Heatmaps of the expression of the 50 top-ranked genes: (**A**) negatively or (**C**) positively correlated with the expression of *Brca1 a*cross all samples. Color bars codify the gene expression on a log2 scale. Higher gene expression corresponds to a redder color. Yellow frames mark the samples used to calculate the correlations. (**B**) Bar plots of the gene-expression correlation with *Brca1*. The central panel depicts the correlation of the expressions of all genes with the expression of *Brca1*. Left and right panels represent the correlation of the top 50 negatively- and positively-correlated genes, respectively. Green and red colors represent negatively- and positively-correlated genes, respectively. (**D**) Heatmap of the expression of the known Brca1-related miRNAs [24]. T: miRNAs targeting Brca1, U: miRNAs up-regulated by Brca1, D: miRNAs down-regulated by Brca1. (**E**) Violin plots of the expression distribution of the Brca1-related miRNAs. Black crosses represent mean positions. Black points represent the spread of the expression of the genes used to build the distributions. The samples are denoted with E, I, and K for endothelial, Ito, and Kupffer cells, C and T for control and TME cells, and TP and TM denote the CRC primary and tumor liver metastasis cells, respectively. For each mRNA sample, we used 4 biological replicates, and for each miRNA sample 3 biological replicates, except 2 for ECs and KTs.

Since *Brca1* was found to be up-regulated in all TME cells, we performed correlation analysis to find genes with expression highly positively or negatively related to the *Brca1* expression across all of the samples of Figure 4A–C. *Slurp1* and *Acnat2* were amongst the top highly positively correlated genes identified in Figure 4C. Slurp1 is a member of the Ly6/uPAR family and reduces the proliferation of human colorectal adenocarcinoma HT-29 cells [18]. Acnat2 is the mouse homologue of BAAT (bile acid-CoA:amino acid N-acyltransferase) and defects in BAAT have been identified as a cause of familial hypercholanemia [19].

*Tpt1*, tumor protein translationally-controlled 1, also known as p23, fortilin, or histamine releasing factor (HRF), plays a role in carcinogenesis, is up-regulated in some cancer cells, and promotes colorectal cancer invasion and metastasis [20]. *Rnf167*, or *Ring105*, is an E3 ubiquitin ligase, and along with UbcH6 regulates the tumor suppressor TSSC5 in Wilms’ tumor, rhabdomyosarcoma, and hepatoblastoma [21]. The complex UbcH6-Rnf167 is a ubiquitin-proteasome pathway that targets TSSC5 [21] and is implicated in the regulation of cellular endosome trafficking [22]. Emd is a member of the nuclear lamina-associated protein family acting as an enhancer of autophagosome formation in the C16-ceramide autophagy pathway in colon cancer [23].

We next checked for the expression of *Brca1*-related miRNAs across all the liver samples. With the exception of *miR-17*, *miR-146b*, and *miR-182*, all of the *Brca1*-related miRNAs were strongly dysregulated in all the liver samples Figure 4D,E. All of the dysregulated miRNAs, *miR-155*, were down-regulated in TME endothelial and Ito cells, but up-regulated in TME Kupffer cells independently of the putative regulation pattern previously described [24].

### 3.5. The Brca1-Centered Gene-miRNA-Protein Network Shows miR-212-3p as Simultaneously Down-Regulated in Tumor-Colonized Samples

**Figure 5:**
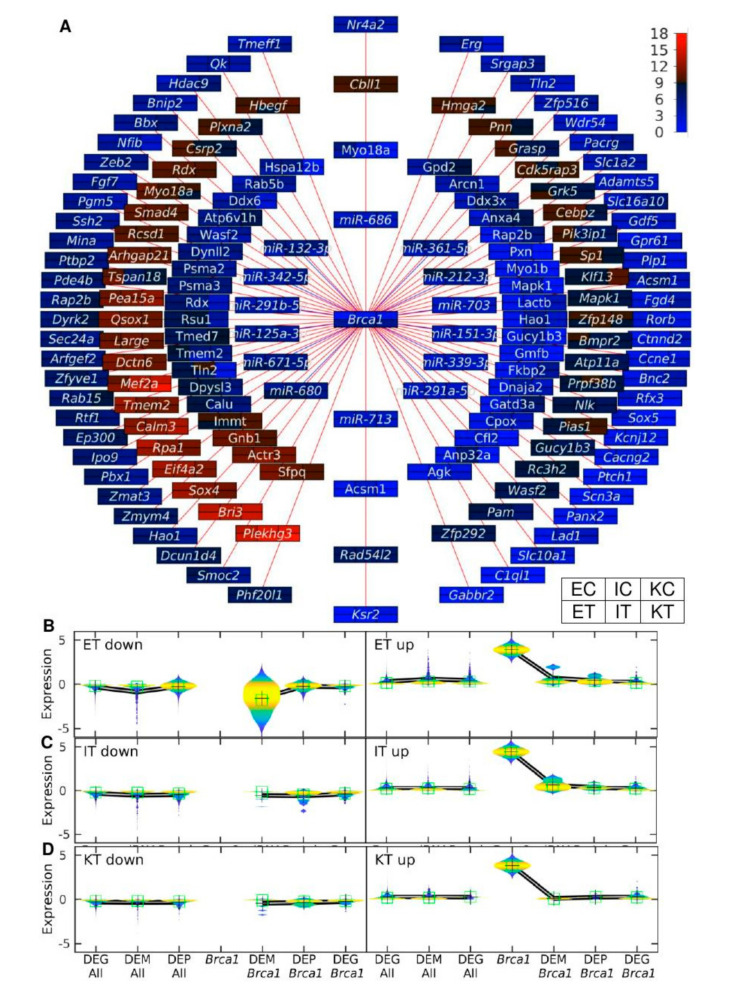
Proposed gene-miRNA-protein network centered in *Brca1*. (**A**) The network center is the gene expression level of *Brca1*. The concentric rings from the center towards the periphery present the expression level of the miRNAs targeting *Brca1*, the protein level of the proteins targeted by such miRNAs, and the expression level of the genes targeted by such miRNAs (the last ring is double), respectively. Each molecule (gene, miRNA, protein) is represented by a rectangle with six colors codifying the expression of the samples in the order given by the legend frame. Higher gene expression corresponds to redder color. Violin plots in endothelial (**B**), Ito (**C**), and Kupffer (**D**) of the distribution of the down- and up-regulated molecules in tumor vs. the control of DEGs, DEMs, and DEPs in all the data and in *Brca1*, miRNA targeting *Brca1*, in proteins, and in genes targeted by such miRNAs. For each mRNA sample, we used 4 biological replicates; for each miRNA sample, 3 biological replicates, except 2 for ECs and KTs; for the proteomics data, 9 biological replicates for ECs, 7 for ETs, 6 for ICs and ITs, and 5 for KCs and KTs.

To investigate the gene-miRNA-protein interaction involved in the up-regulation of *Brca1* in the TME samples we created networks depicting the expression of the miRNAs, proteins and genes in consecutive rings around *Brca1* Figure 5A. This shows a high degree of correlation in the expression levels of the different molecules that interact with Brca1 in all the different cell types analyzed. For example in the miRNAs ring, *miR-212-3p* was found to be down-regulated in tumors. In the protein ring, we observed that Gatd3a was Kupffer-specific, whereas Anp32a, Gucy1b3, and Hao1 were Ito-specific, and Calu more highly expressed in endothelial cells. We observed a set of genes that were highly expressed in all the liver samples (*Pea15a*, *Qsox1*, *Large*, *Dctn6*, *Mef2a*, *Tmem2*, *Calm3*, *Rpa1*, *Eif4a2*, *Sox4*, *Bri3*, and *Plekhg3*). In addition, we studied the distribution of the differential expressions between tumor and control samples of the molecules involved in the network using violin plots Figure 5B–D. For each molecule type, we split the distribution between down- and up-regulation in tumor vs. control samples. We performed the analysis for *Brca1* Expression, DEM targeting *Brca1*, and DEG and DEP of other genes and proteins targeted by the miRNAs that target *Brca1*. As a control with which to compare these distributions, we used the distributions of all the DEM, DEG, and DEP associated with molecules not targeted by miRNA that target *Brca1*. The analysis was performed for differentially expressed molecules in ET vs. EC in Figure 5B, IT vs. IC in Figure 5C, and KT vs. KC in Figure 5D. We observed that miRNAs targeting *Brca1* were generally down-regulated in tumor endothelial cells.

### 3.6. RT-PCR Analysis Validated that the Brca1-Related miR-212-3p Is Differentially Expressed between Control and Tumor-Colonized Samples

We performed RT-PCR analysis to validate four of the miRNAs simultaneously down-regulated in the three cell types in the TME (i.e., *miR-21a*, *miR-29a*, *miR-16* and *miR-146a* Figure 2D,E) along with *miR-212-3p* that has previously been associated with *Brca1* expression Figure 5A. RT-PCR measurements are depicted similarly to microarray results in Figure S4. RT-PCR validated the results on miR-16 in Ito cells, with lower expression in tumor-colonized cells in relation to the control, a finding in agreement with reports on the down-regulation of miR-16 in other cancers such as cancers of the breast [25], colon [26,27], brain [28,29], lung [30], lymphatic system [31,32,33,34], ovaries [35], pancreas [36] and prostate [37]. RT-PCR analysis further validated that the *Brca1-*related miRNA *miR-212-3p* is indeed differently expressed between the control and tumor samples.

### 3.7. BRCA1 Is the Last BASC Gene Activated in the TME

**Figure 6:**
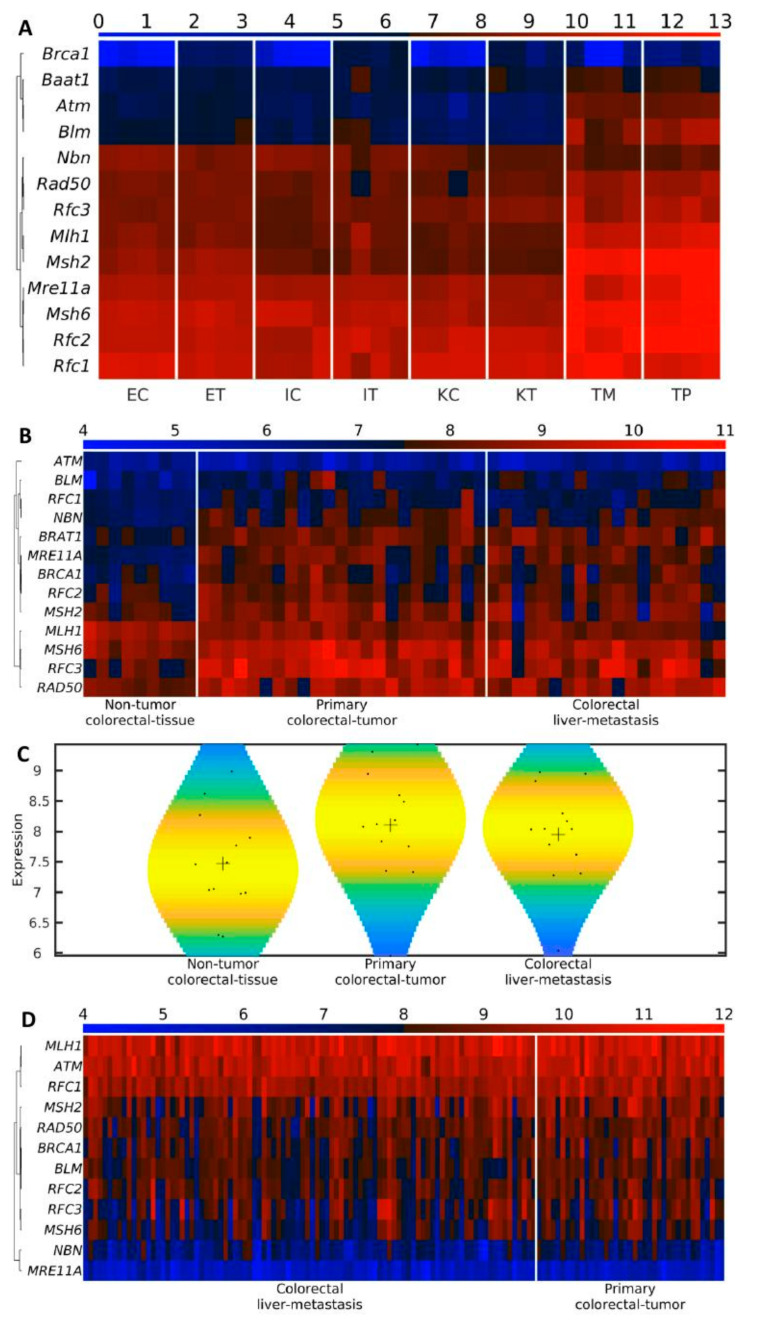
*BRCA1* is the last BASC gene activated in TME. Heatmap of the expression of BASC-related genes in (**A**) mouse liver cell samples. The samples are denoted with E, I, and K for endothelial, Ito, and Kupffer cells, C and T for control and TME cells, and TP and TM denote the CRC primary and tumor liver metastasis cells, respectively. Higher gene expression corresponds to a redder color (for each mRNA sample, we used 4 biological replicates); and (**B**) human non-tumor colorectal tissue, primary colorectal tumor, and colorectal liver metastasis from 51 patients [39]. Higher gene expression corresponds to a redder color. (**C**) Violin plot of the expression distribution of human BASC-related genes. Black crosses represent mean positions. Black points represent the spread of the expression of the genes used to build the distributions. (**D**) Heatmap of the expression of BASC-related genes in human primary colorectal tumor and colorectal liver metastasis from 133 patients [40]. Higher gene expression corresponds to a redder color.

To understand the special role of BRCA1 in colorectal liver metastasis, we analyzed the expression of the genes of the BASC (BRCA1-associated genome surveillance complex), a super complex of BRCA1-associated proteins involved in the recognition and repair of aberrant DNA structures. BASC includes the tumor suppressors and DNA damage repair proteins MSH2, MSH6, MLH1, ATM, BLM, the RAD50-MRE11-NBS1 protein complex, and the DNA replication factor C (RFC), a protein complex facilitating the loading of the Proliferating Cell Nuclear Antigen (PCNA) onto DNA [38]. In all analyzed mouse liver cells (control and TME), almost all the members of BASC were highly expressed. The exceptions were *Blm*, *Rad50*, and *Atm*, which were not highly expressed, with progressively decreasing expression from Endothelial→Ito→Kupffer. Interestingly, *Brca1* itself displayed very low expression in all control samples but was highly expressed in all TME samples in Figure 6A. In the tumor samples (TM and TP), all of the BASC genes displayed increased expression, suggesting that *Brca1* is the last BASC gene to become activated in the TME samples.

To further investigate the behavior of BASC genes in CRC, we downloaded two independent datasets with transcriptomics data of liver metastasis from the GEO database. In the dataset with non-tumor colorectal tissue, primary colorectal tumor, and colorectal liver metastasis from 51 patients [39] (GSE81558), we found that *ATM* is middle-expressed in the three types of samples, around a third of the BASC genes (*MLH1*, *MSH2*, *MSH6*, *RAD50*) are highly expressed in the three types of samples, and more than the half (*BLM*, *BRAT1*, *BRCA1*, *MRE11A*, *NBN*, *RCF1*, *RFC2*, *RCF3*) are up-regulated in cancer samples (primary colorectal tumor and colorectal liver metastasis) in relation to the non-tumor colorectal tissue in Figure 6B. Thus, generally, the BASC gene expression is higher in cancer than in non-cancer samples in Figure 6C. In the dataset with primary colorectal tumor and colorectal liver metastasis from 133 patients [40] (GSE41568), *MRE11A* and *NBN* are low-expressed in primary colorectal tumor and colorectal liver metastasis, *MLH1*, *ATM*, and *RFC1* and homogeneously high-expressed in all the samples, and the rest of the BASC genes, including *BRCA1*, are high-expressed, with some variation between patients, *MSH6* and *MHSH2* being the highest and lowest variable ones, and *BRCA1* being high-expressed with a middle variability among patients in Figure 6D. These results support the hypothesis that *BRCA1* is the last BASC gene activated in colorectal liver metastasis.

### 3.8. The Most Probable Sequence of Cell Activation during Metastasis is Endothelial→Ito→Kupffer

To investigate the sequence of cell activation across the three cell types (endothelial, Ito, Kupffer) during metastasis, we searched for genes continuously up- or down-regulated in the three possible sequences of activation of these three cell types in TME cells Table 1 and in control cells Table 2, using a fold–change of 2 (in log_2_ scale). In the sequence activation of TME cells, we found 34 transcripts up-regulated and none down-regulated across the Endothelial→Ito→Kupffer sequence in Figure S5A. Interestingly, all of these transcripts were more highly expressed in TME cells than in control cells in Figure S5B, whereas the distribution of the expression of all the transcripts was the same for all the samples Figure S5C. We found 16 transcripts up-regulated and 11 transcripts down-regulated across the Endothelial→Kupffer→Ito sequence. Out of these 11 down-regulated transcripts, one corresponded to the apelin (*Apln*) gene and all the other 10 probes to the endothelin1 (*Edn1*) gene. It is known that activation of autocrine and paracrine signaling by Edn1 binding to its receptors elicits pleiotropic effects on TME cells and on the host microenvironment. This activation modulates cell proliferation, apoptosis, migration, EMT, chemoresistance, and neovascularization [41]. We also found that the *Edn1* probes were up-regulated in ET in relation to EC. We found only one transcript (*Tm4sf4*) down-regulated across the sequence Ito→Endothelial→Kupffer and none were up-regulated. We used the control cells as reference for the sequence of cell activation in Table 2. We found almost no transcripts continuously up- or down-regulated in any cell activation sequence; only 4 in the up-regulation of the Endothelial→Ito→Kupffer control sequence, 6 in the up-regulation of the Ito→Endothelial→Kupffer control sequence, and 1 in the down-regulation of the Ito→Endothelial→Kupffer control sequence. The sequence of cell activation during metastasis with most transcripts continuously up-regulated is the Endothelial→Ito→Kupffer sequence of TME cells. Interestingly, the transcripts up-regulated in the TME sequence were also up-regulated in the TME cells compared to their control cell counterparts. Thus, there appears to be a cascade of genes that progressively increase their expression from Endothelial to Kupffer cells through the Ito cells, and simultaneously are more expressed in TME than in control cells. Taking these results together, the observed sequence of cell activation during metastasis is most likely Endothelial→Ito→Kupffer.

### 3.9. Multiple Inflammation-Related Genes Are Up-Regulated in TME Samples

Among the commonly up-regulated genes identified in TME was Sp110, a member of the SP100/SP140 family of nuclear body regulating genes involved in the inflammatory response [17]. We checked the behavior of the inflammation-related genes [42] in our samples in the context of the CRC pre-metastatic niche. Several of these inflammation-related genes are members of the SP100 family, and many of them, such as *S100a4*, *S100a8*, and *S100a9*, were up-regulated in all the TME samples in relation to the control. Also, the orosomucoids 1 and 2 (*Orm1* and *Orm2*) that encode key acute phase plasma proteins classified as acute-phase reactants whose expression is increased due to acute inflammation were up-regulated in Ito and Kupffer TME (Figure S6). Interestingly, with the exception of *S100a4*, these genes remain very low-expressed in primary CRC tumors (TM) and in liver metastasis (TM).

### 3.10. Immunohistochemical Analysis of TMAs from Human CRC Liver Metastases Reveals that BRCA1-Positive Samples Correlate with CRC Stage

To study the clinical relevance of BRCA1 in the metastasis of human CRC in the liver, we performed Tissue Microarrays (TMAs) with liver metastatic samples from 34 CRC patients. Following previously reported classifications [43], we stratified liver metastatic samples into two groups: metastases detected within six months from time of diagnosis, known as synchronous, and metastases that debuted six months or years later after the time of diagnosis, known as metachronous. In the first group, we included 11 metastases from Stage IV CRCs, while in the second group (*n* = 23), we included metastases from primary CRCs that were previously diagnosed as Stage I (*n* = 4), Stage II (*n* = 10), and Stage III (*n* = 9).

**Figure 7:**
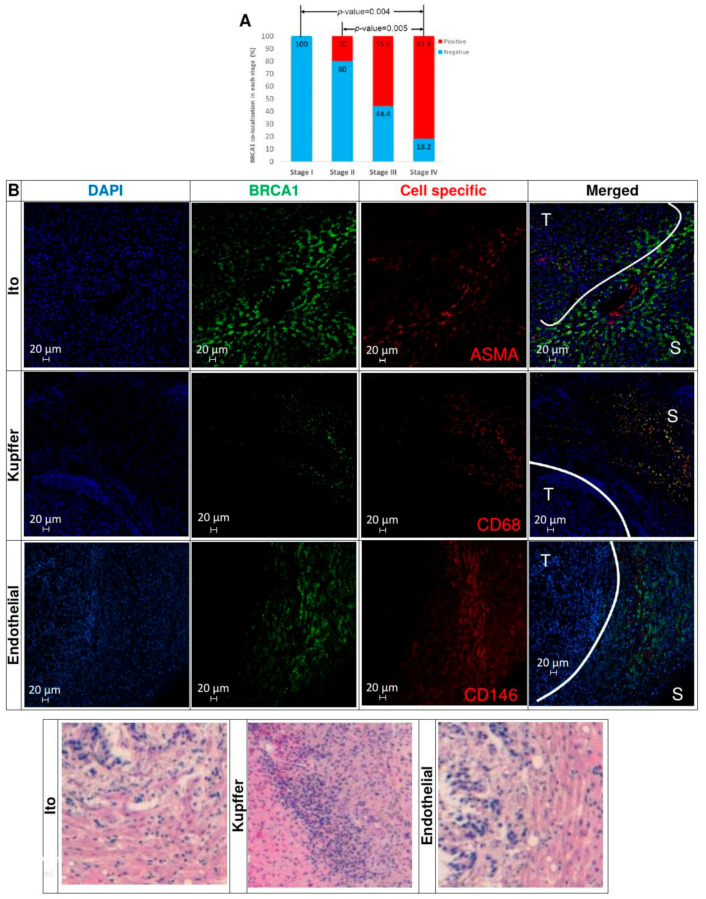
BRCA1 co-localization in TME cells from human CRC liver metastases. (**A**) The percentage of simultaneous BRCA1 expression in endothelial, Ito, and Kupffer cells significantly increases in metastases from Stage IV tumors (synchronous) compared to Stage I and II ones (metachronous). Positive = Co-expression of BRCA1 protein in the three TME cell types. Negative = No expression of BRCA1 in any TME cell type. (**B**) BRCA1 expression is co-localized on Ito, Kupffer, and endothelial cells from liver metastases from CRC categorized as Stage IV at first diagnosis (synchronous). White lines delimit the tumor cell tissue regions (T) from the non-tumoral cell stromal area (S). The last row shows the hematoxylin and eosin staining images.

**Figure 8:**
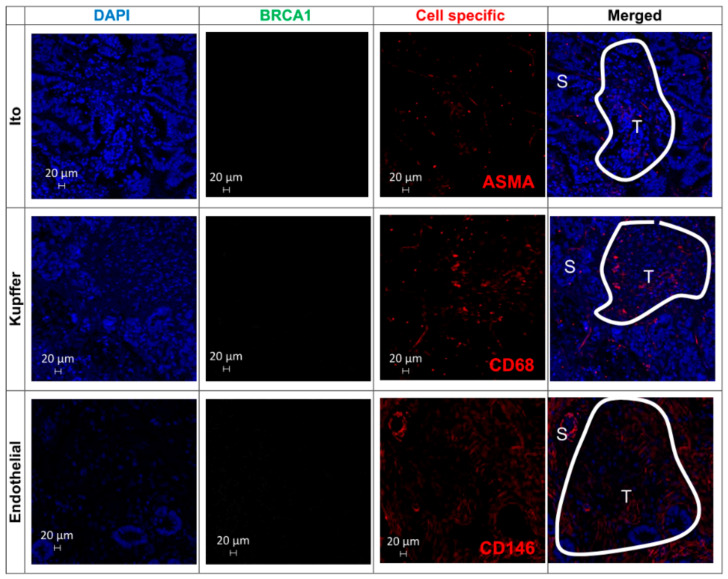
The BRCA1 protein is not expressed in all the TME cells from CRC liver metastases from Stage I patients (metachronous). BRCA1 expression on TMA samples on Ito, Kupffer, and endothelial cells from human CRC liver metastases. White lines delimit the tumor cell tissue regions (T) from the non-tumoral cell stromal area (S).

BRCA1 protein was simultaneously detected in Ito, Kupffer, and endothelial cells in the TME from the 81.8% of synchronous metastases (Stage IV). However, none of the metachronous metastases originated from Stage I CRCs expressed BRCA1 in the TME cells, and only 20% of metastases from Stage II primary tumors showed co-localized expression of this protein in the three liver cell types. This co-localization correlates significantly with the moment of occurrence of these metastases (χ^2^ test *p*-value = 0.007) in Figure 7A. Thus, BRCA1 is “turned on” simultaneously in the three cell types of the TME in liver metastases from the most invasive, metastasizing most “early” CRCs in Figure 7B, and BRCA1 protein is not expressed, i.e., it is “turned off” in all host cells of metachronous metastases arising from Stage I primary CRCs in Figure 8. Higher magnification and resolution images showing the subcellular localization of BRCA1 and its co-localization with cell markers in metastatic lesions are presented in Figure S7.

## 4. Discussion

Previous studies on BRCA1 have focused on its regulatory function in epithelial cells and tumors arising from these cells, the best-known being breast cancer [44]. Here, we obtained for the first time a general overview of the omics deregulation of the liver metastasis microenvironment analyzing liver sinusoidal endothelial, Ito, and Kupffer cell changes during CRC colonization of the liver. Although the primary tumor creates a special environment and changes the surrounding cell functions, provoking angiogenic support, the metastatic tumor has been also studied as a creator of the TME [45,46]. The liver, as one of the most metastasized organs, is not an exception, and the metastasis TME has been studied from various viewpoints. Liver endothelial cells were described as the first cells interacting with tumor cells and consequently the first cells suffering the phenotypic changes. Over-expression of integrins such as β2 facilitates tumor implantation and progression [47]. Ito cells, a cell type resident in the Disse space, completely change their phenotype to myofibroblastic cells, which in turn produce extracellular matrix changes and overexpressing matrix metalloproteinases that change tissue architecture and help tumor motility [6]. The resident macrophages of the liver, known as Kupffer cells, after the tumor signals activations, produce inflammatory cytokines suitable for the metastatic progression [48].

While previous studies focused on a unique cell type, obviating the interaction of the whole TME and the different host cells, we simulated the whole liver metastasis TME, allowing us to study each cell type in the complex network interactions at the omics scale.

At the three analyzed molecular levels (gene, miRNA, and protein), the Kupffer cells show the highest level of resilience to cancer penetration, while the endothelial cells have the lowest, an observation that correlates with the chronological cell interaction. The endothelial cells are the first hepatic cells in contact with the tumor cells and suffer the initial cell phenotypic transformation [49]. The interaction of the two cell types allows the tumor-cell anchorage to the endothelium and extravasation to the liver parenchyma, triggering the inflammatory cascade involving Ito and Kupffer cells [50].

The low correlation between protein and gene expression could be due to the high level of miRNA regulation, indicated by the enrichment of lower-expressed proteins in the region of middle-expressed genes that could be targeted by miRNAs. *miR-16*, the only miRNA down-regulated in TME endothelial and Ito cells but up-regulated in TME Kupffer cells, is among the miRNAs described as targeting *Brca1* [24].

The commonly up-regulated gene in all TME cells, *BRCA1*, encodes a nuclear phosphoprotein involved in maintaining genomic stability, DNA repair of double-stranded breaks, tumor suppression and in alternative non-homologous end-joining at uncapped telomeres [51]. The encoded protein combines with other tumor suppressors, DNA damage sensors, and signal transducers, forming the BASC complex. Here, we found that *BRCA1* is the last BASC gene activated in the TME. Mutations in *BRCA1* are responsible for approximately 40% of inherited breast cancers and more than 80% of inherited breast and ovarian cancers.

Although BRCA1 mutation is commonly associated with cancer susceptibility, BRCA1 appears also as a key player in physiological situations. BRCA1 expression in lymphocytes varies during the day with higher diurnal amount and depletion during the night, as a response of the cells to the repair mechanism against aggressions and cell wear [52]. BRCA1 was also described as a protector factor in fibroblast exposed to tumor factors avoiding cell malignancy [53]. The role of BRCA1 as a protector against extracellular insults in an inflammatory context during hepatic metastasis was also evidenced in endothelial cells [54]. During metastasis progression, inflammatory molecules and reactive oxygen species concentration increases and damages cell components such as organelles and the nucleus, including the DNA [55]. The response of host-organ cells against the inflammation and high reactive oxygen strain concentration is the expression of protective proteins such as Nrf2 [56].

The equal up-regulation of *BRCA1* in endothelial, Kupffer, and Ito cells hints at the pivotal role of *BRCA1* as being responsible for the TME in the liver metastases, a role of clinical relevance since liver metastases from patients with CRC express the BRCA1 protein in the three types of TME cells depending on the time of onset of the metastases. Synchronous liver metastases show simultaneous expression of BRCA1 protein in the host cells, whereas metachronous metastases from primary CRCs that were previously diagnosed as non-advanced did not express BRCA1 in these cells. Thus, the simultaneous expression of BRCA1 in endothelial, Kupffer, and Ito cells occurs in tumors that have more quickly invaded the large intestine wall, locoregional lymph nodes, and the liver, i.e., the BRCA1 gene and protein are “turned on” in three key cells types of the liver TME in early-invading tumors but not in late-invading CRCs.

It is difficult to conclude whether the high rate of BRCA1 co-expression in TME is a cause or consequence of the rapid onset of CRC metastases in the liver. In the context of previous studies, it could be inferred that this up-regulation is due to a protective reaction of host cells in the presence of a high hypoxic and inflammatory status detected in rapidly growing tumors [50,52,54,56]. Nevertheless, a pro-tumor role of BRCA1 in TME in the early occurrence of liver metastases cannot be discarded. Studies in primary CRCs and hepatocarcinoma demonstrated that the subcellular location of BRCA1 is associated with a different prognosis, better when the expression is cytoplasmic and worse when it is nuclear [57]. Also, epigenetic changes of *BRCA1* induce a CAF-like state in fibroblasts from healthy tissues, increasing cancer predisposition [58]. Besides, it is known that endothelial, Ito, and Kupffer cells express many cell-surface markers and secrete different soluble factors associated with different steps of metastatic invasion into the liver [48,58,59]. An additional contribution in solving this conundrum is our finding in the Brca1-centered gene-miRNA-protein network that *miR-212-3p* is simultaneously down-regulated in tumor-colonized samples, which we validated by RT-PCR. Interestingly, overexpression of miR-212 inhibits liver and lung metastasis by targeting MnSOD and prevents tumor progression [60].

## 5. Conclusions

In this work, we performed an integrative omics analysis of liver TME dysregulation in gene, miRNA and protein expression comparing healthy and tumor colonized liver sinusoidal endothelial, Ito and Kupffer cells by colorectal cancer cells. Among many dysregulated genes we detected simultaneous overexpression of BRCA1 in all the analyzed hepatic TME cells types. We build a network of dysregulated miRNAs that could interact with BRCA1 to up regulate its expression in the liver TME. To validate the role of BRCA1 in human liver metastasis biopsies, were the expression of BRCA1 in the three cell types was correlated with advanced tumor stage. Further studies on subcellular location of BRCA1, mutations and methylation status of *BRCA1* gene of host cells from liver metastases, and the involvement of these geno- and phenotypic simultaneous changes in metastatic phases, are needed to shed light to the molecular mechanism of BRCA1 in liver metastasis.

## Figures and Tables

**Table 1:** Cell activation sequence in the tumor cells (fold change 2 in log_2_ scale). For each mRNA sample, we used 4 biological replicates.

Cell Activation Sequence	*R*	*N*	Genes
Endothelial→Ito→Kupffer	↑	34	*3110047P20Rik, AW060742, A_55_P1964173, A_55_P2091525, Abca17, Armcx3, Atp8a1, B930078G14Rik, Cd300a, Elavl4, Fam160a2, Fdxacb1, Fnbp1, Gm10914, Golga7b, Med13, Nhlh1, Npcd, Rdh1, Slc1a4, TC1648127, Zfp133-ps, chr10:69842010-69842465_F, chr11:53574182-53574644_R, chr14:78214602-78225827_F, chr17:17462450-17488725_R, chr1:138442536-138521080_F, chr1:182686731-182709331_F, chr4:131905739-131910814_R, chr5:74340084-74346709_R, chr6:136440100-136450050_F, chr6:146493273-146493932_R,* *chr8:35143801-35144482_F, chrX:120300373-120302404_F*
	↓	0	
Endothelial→Kupffer→Ito	↑	16	*1300017J02Rik, Aadac, Afm, Ahsg, Apof, C8a, C8b, Cyp2a12, Cyp2d10, Cyp2d26, Habp2, Itih, Itih2, Serpinc1, Serpind1, Ugt2b36*
	↓	11	*Apln, Edn1* (10 probes)
Ito→Endothelial→Kupffer	↑	0	
	↓	1	*Tm4sf4*

*R* is the type of regulation across the cell sequence: ↑ up-regulation, ↓ down-regulation. *N* is the number of transcripts continuously up- or down-regulated across the cell sequence.

**Table 2:** Cell activation sequence in the control cells (fold change 2 in log_2_ scale). For each mRNA sample we used 4 biological replicates.

Cell Activation Sequence	*R*	*N*	Genes
Endothelial→Ito→Kupffer	↑	4	*Igf2bp, Prss46, Slc6a4, Tnfsf4*
	↓	0	
Endothelial→Kupffer→Ito	↑	0	
	↓	0	
Ito→Endothelial→Kupffer	↑	6	*Apob, Beta-s, Clec4b1, Hba-a2, Hbb-b1, Hbb-b2*
	↓	1	*Apol10b*

*R* is the type of regulation across the cell sequence: ↑ up-regulation, ↓ down-regulation. *N* is the number of transcripts continuously up- or down-regulated across the cell sequence.

## Data Availability

The data discussed in this publication have been deposited in NCBI’s Gene Expression Omnibus (GEO) [61] and are accessible through GEO Series accession number GSE156431 (https://www.ncbi.nlm.nih.gov/geo/query/acc.cgi?acc=GSE156431). The mass spectrometry proteomics data have been deposited to the ProteomeXchange Consortium via the PRIDE [62] partner repository with the dataset identifier PXD020913.

## Supplementary Materials

The following are available online at https://www.mdpi.com/2072-6694/12/9/2380/s1. Figure S1: Pairwise scatter plots of mRNA, miRNA and protein expression, Figure S2: Differentially expressed proteins between control and TME cells, Figure S3: Correlation between gene and protein expression, Figure S4: Heatmap of the RT-PCR expression of the selected miRNAs in Ito and Kupffer cells, Figure S5: The sequence of cell activation during metastasis seems to be Endothelial→Ito→Kupffer, Figure S6: Multiple inflammation-related genes are up-regulated in TME samples, Figure S7: BRCA1 expression co-localization in TME cells from CRC liver metastases.

## Short Summary

Integrative omics study of expression of genes, miRNAs and proteins in three types of mouse liver cells from the TME of CRC liver metastasis revealed that *Brca1* is simultaneously up-regulated in all the TME cells. Further immunohistochemical analysis in human liver metastases showed that BRCA1 protein is co-localized in the three cell types from 81.8% of synchronous metastases from Stage IV CRC.

## Computer Code and Software

Our newly developed software package miRDiana to search for miRNA target genes is freely available at https://sourceforge.net/projects/mirdiana/

## Abbreviations

BASC	BRCA1-Associated genome Surveillance Complex
CRC	Colorectal Cancer
DEG	Differentially Expressed Gene
DEM	Differentially Expressed MicroRNA
DEP	Differentially Expressed Protein
EC	liver sinusoidal endothelial cell (E) from healthy/control (C) mice
ET	liver sinusoidal endothelial cell (E) from Tumor microenvironment (T)
IC	Ito cell (I) from healthy/control (C) mice
IT	Ito cell (I) from Tumor microenvironment (T)
KC	Kupffer cell (K) from healthy/control (C) mice
KT	Kupffer cell (K) from Tumor microenvironment (T)
miRNA	microRNA
TM	Tumor liver Metastasis
TME	Tumor Microenvironment
TMA	Tissue Microarray
TP	CRC Primary Tumor
