# Supplementary material for: An Integrative Omics Approach Reveals Involvement of BRCA1 in Hepatic Metastatic Progression of Colorectal Cancer
Source: Cancers (Basel). 2020 Aug 22;12(9):2380. doi: 10.3390/cancers12092380 (PMC7565528; doi:10.3390/cancers12092380)
Supplement: Supplementary file 1 [file cancers-12-02380-s001.pdf]

Supplementary Materials:

An Integrative Omics Approach Reveals That Brca1 Is Involved in Hepatic Metastatic Progression of Colorectal Cancer

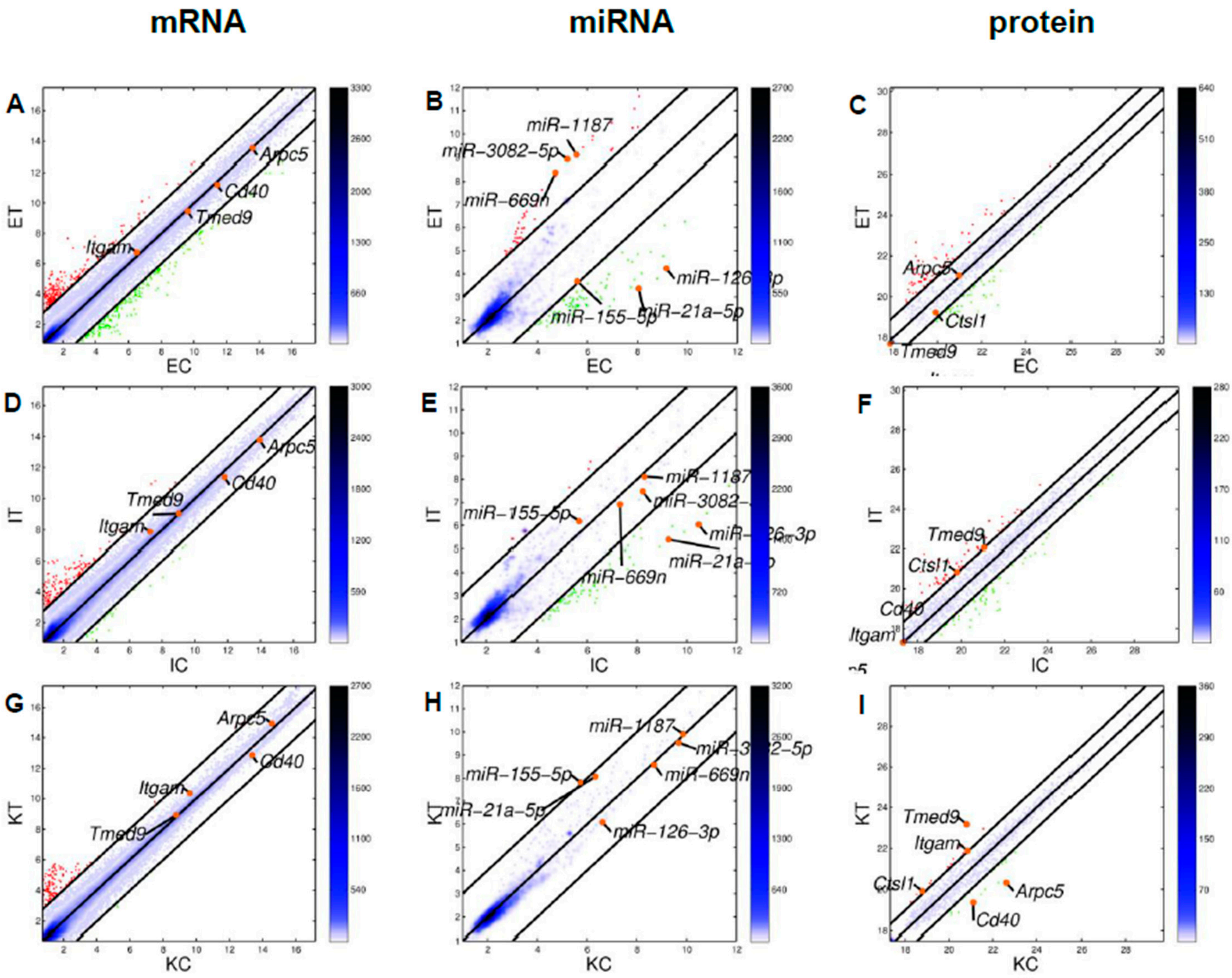

Figure S1. Pairwise scatter plots of mRNA, miRNA and protein expression. Three types of liver cells were collected, namely liver sinusoidal endothelial cells (E), Ito cells (I) and Kupffer cells (K), from control (C) and TME (T), and isolated to perform omics experiments (gene expression, miRNA expression microarrays and proteomics). The black lines are the boundaries of the 2-fold changes in the expression levels between the paired samples. Molecules up-regulated in ordinate samples compared with abscissa samples, are shown with red dots; those down-regulated, with green. Some marker positions are shown as orange dots. The color bar indicates the scattering density. Darker blue color corresponds to higher scattering density. Expression levels are log_2 scaled.

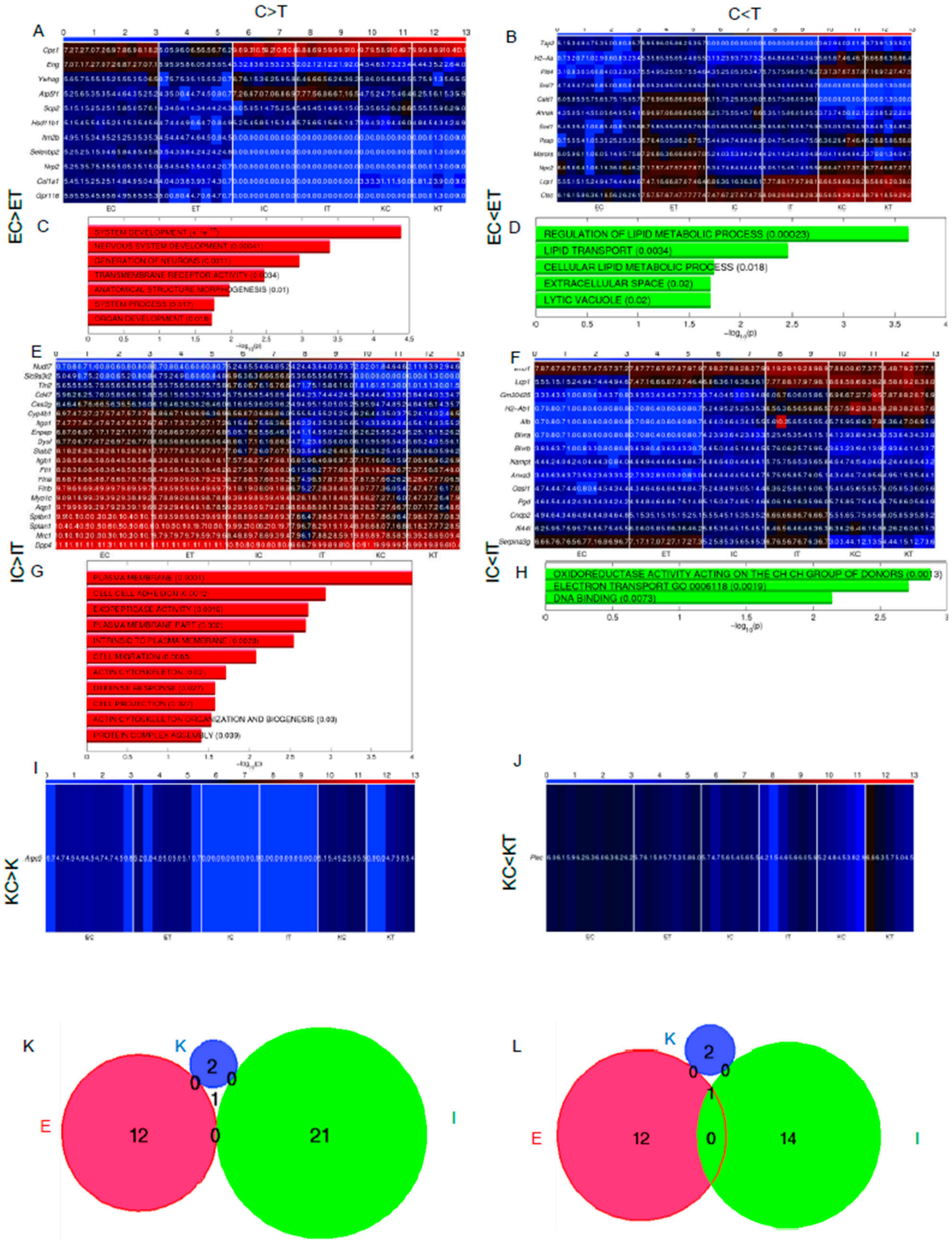

Figure S2. Differentially expressed proteins between control and TME cells. The results for TME down- (C>T) and up- (C<T) regulated in relation to the control are given in the left and right column, respectively. Heatmaps of the expression of the top ranked TME DEPs, in decreasing order of significance, (**A**) down- and (**B**) up- regulated in endothelial cells. The color bar codifies the protein expression in $\log_2$ scale. Higher protein expression corresponds to redder color. Bar plots of the $-\log_{10}$(*p*-value) of the significant enriched GO terms of the TME DEPs (**C**) down- and (**D**) up- regulated in endothelial cells. Longer bars

correspond to higher statistical significance of the enrichment (*p*-values inside parentheses). Heatmaps of the expression of the top ranked TME DEPs in decreasing order of significance (**E**) down- and (**F**) up- regulated in Ito cells. The color bar codifies the protein expression in $\log_2$ scale. Higher protein expression corresponds to redder color. Bar plots of the $-\log_{10}(p\text{-value})$ of the significant enriched GO terms of the TME DEPs (**G**) down- and (**H**) up-regulated in Ito cells. Longer bars correspond to higher statistical significance of the enrichment (*p*- values inside parentheses). Heatmaps of the expression of the only TME DEP (**I**) down- and (**J**) up- regulated in Kupffer cells. The color bar codifies the protein expression in $\log_2$ scale. Higher protein expression corresponds to redder color. Euler-Venn diagrams of the TME DEPs (**K**) down- and (**L**) up-regulated shared by the endothelial, Ito cells and Kupffer cells. The samples are denoted with E, I and K for endothelial, Ito and Kupffer cells, and C and T, for control and TME cells, respectively.

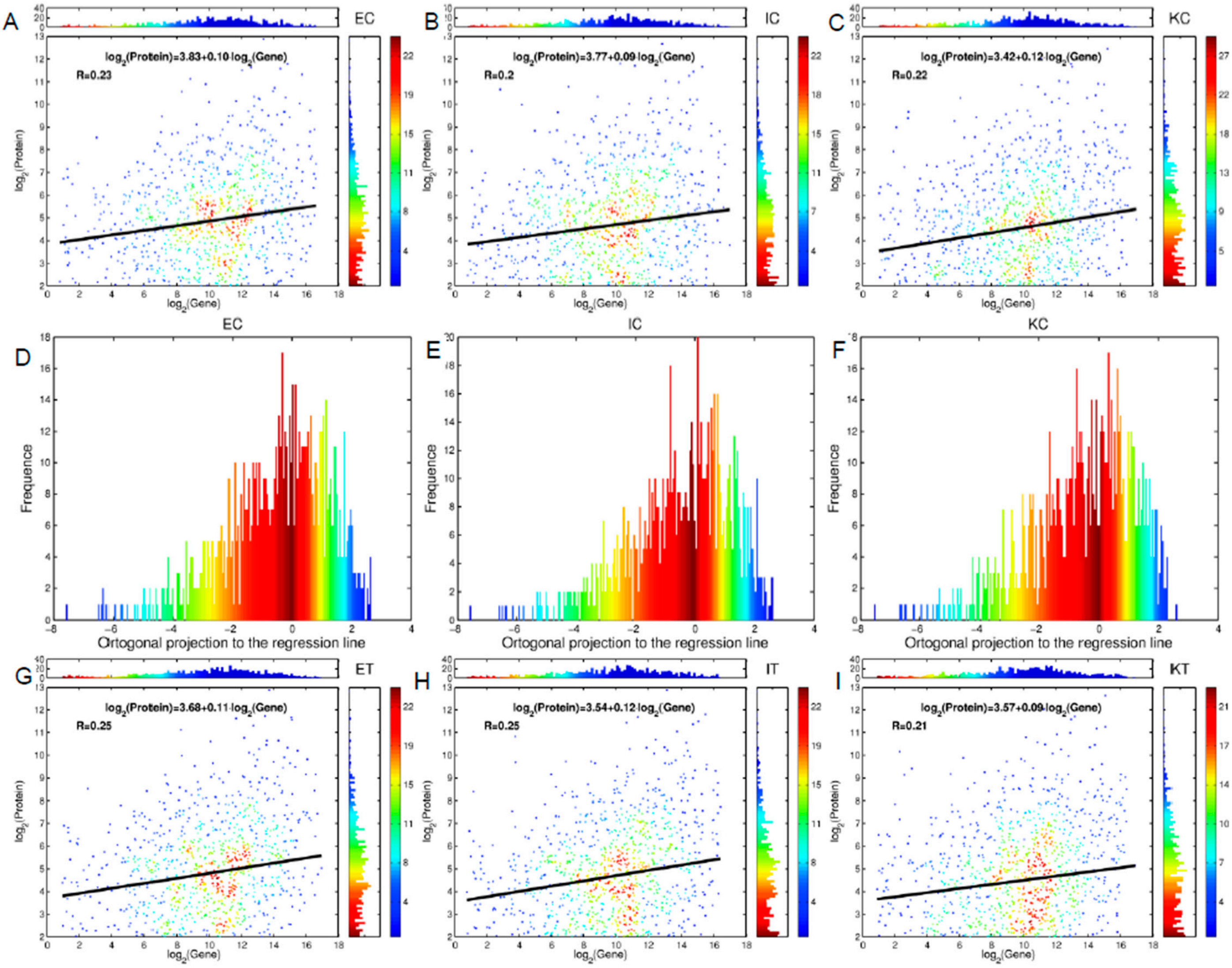
A
EC
log2(Protein)=3.83+0.10·log2(Gene)
R=0.23
B
IC
log2(Protein)=3.77+0.09·log2(Gene)
R=0.2
C
KC
log2(Protein)=3.42+0.12·log2(Gene)
R=0.22
log2(Protein)
log2(Gene)
D
E
F
Frequence
Ortogonal projection to the regression line
G
ET
log2(Protein)=3.68+0.11·log2(Gene)
R=0.25
H
IT
log2(Protein)=3.54+0.12·log2(Gene)
R=0.25
I
KT
log2(Protein)=3.57+0.09·log2(Gene)
R=0.21

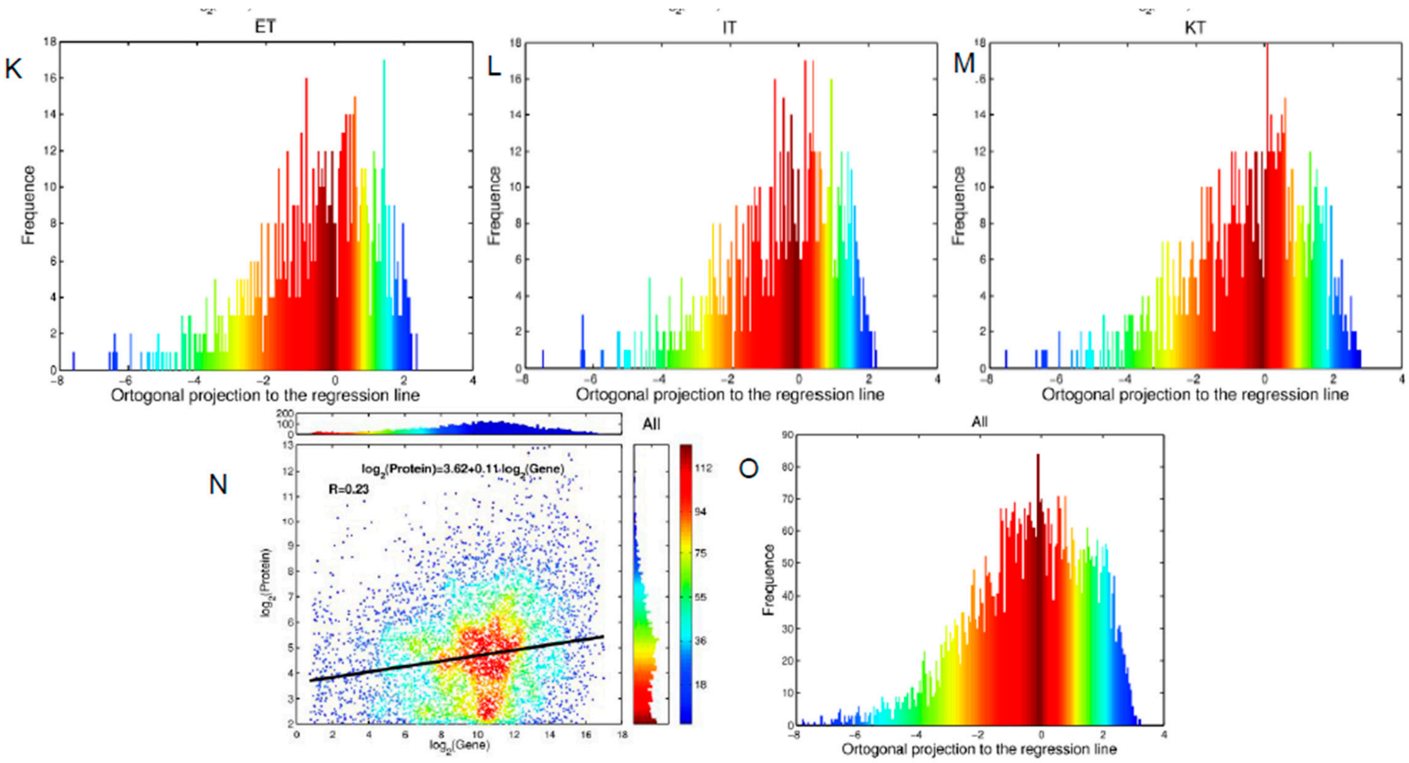

Figure S3. Correlation between gene and protein expression. Endothelial, Ito and Kupffer cells are analyzed in left, central and right column, respectively. Scatter plots of protein expression versus gene expression for (**A-C**) the control cells, and (**G-I**) the TME cells, and (**N**) the merged data of all the cells. The regression line of the protein expression versus gene expression is over imposed in black. R is the Pearson's correlation coefficient between protein and gene expression. Histograms of gene and protein expression are given on the top and to the right of the scatter plots, respectively. The color bars codify the scatter density. Histograms of the projection of the protein – gene points onto the orthogonal to the regression line for (**D-F**) the control cells, (**K-M**) the TME cells, and (**O**) the merged data of all the cells. Higher frequency corresponds to redder color. The samples are denoted with E, I and K for endothelial, Ito and Kupffer cells, and C and T, for control and TME cells, respectively.

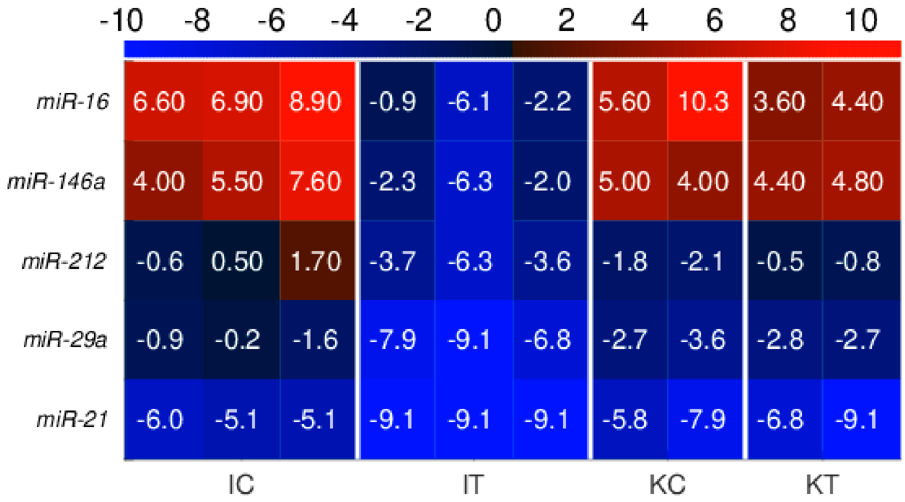

Figure S4. Heatmap of the RT-PCR expression of the selected miRNAs in Ito and Kupffer cells. The color bar codifies the microRNA in $-\Delta C_t$ units (in relation to sno-202). Higher miRNA expression corresponds to redder color.

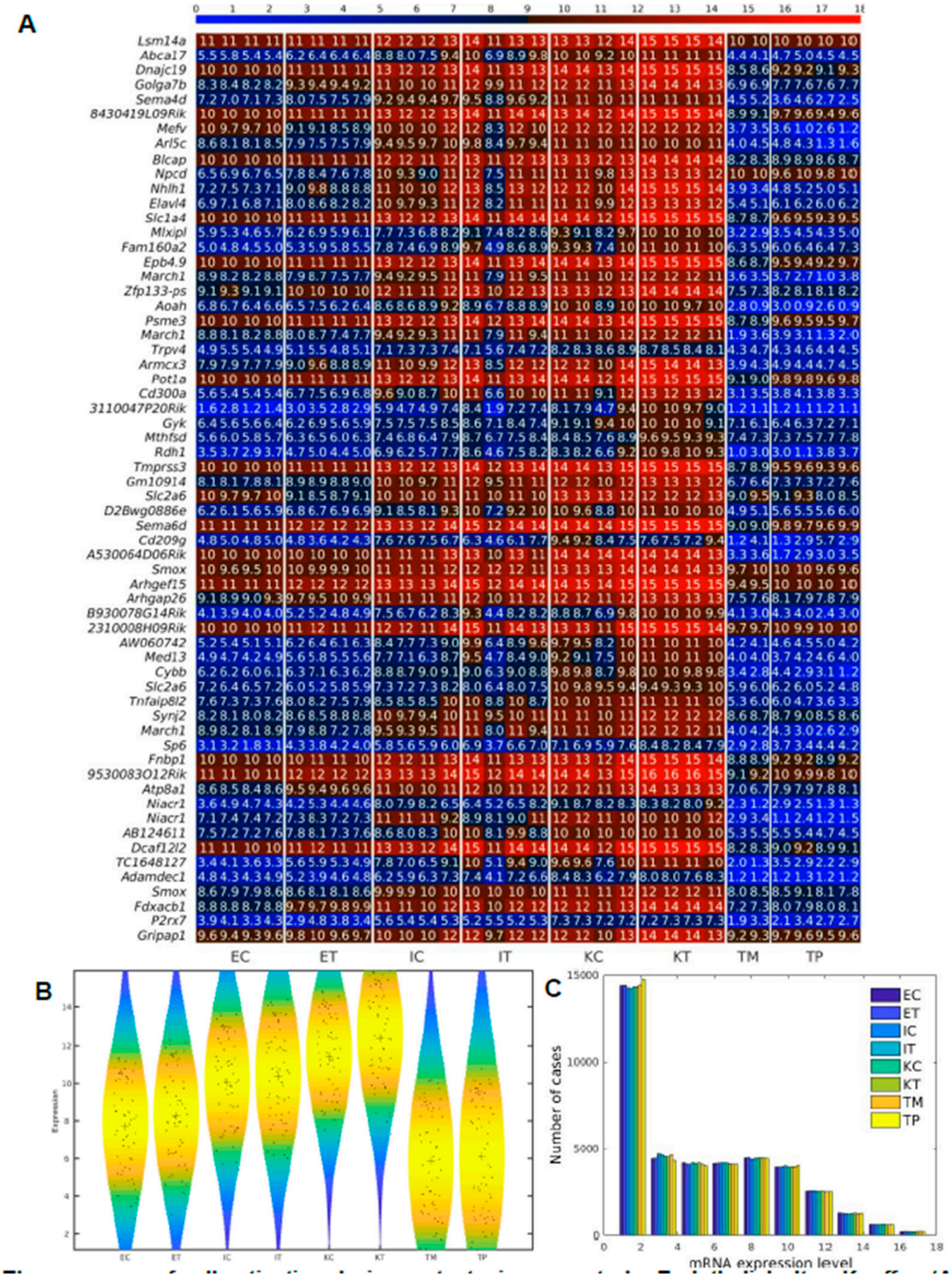

Figure S5. The sequence of cell activation during metastasis seems to be Endothelial→Ito→Kupffer. **(A)** Heatmap of the expression of the continuously up-regulated genes in the TME cells in the activation sequence Endothelial→Ito→Kupffer using a fold change of 2 in $\log_2$ scale. The color bars codify the gene expression in $\log_2$ scale. Higher gene expression corresponds to redder color. (**B**) Violin plot of the expression distribution of the transcripts continuously up-regulated in the TME cells in the activation sequence Endothelial→Ito→Kupffer. The black crosses represent positions of the means. The black points represent the spread of the expression of the genes used to build the distributions. (**C**) Distribution of the number of transcripts expressed for each expression level. The samples are denoted with E, I and K for endothelial, Ito and Kupffer cells, C and T, for control and TME cells, and TP and TM for CRC primary and tumor liver metastasis cells.

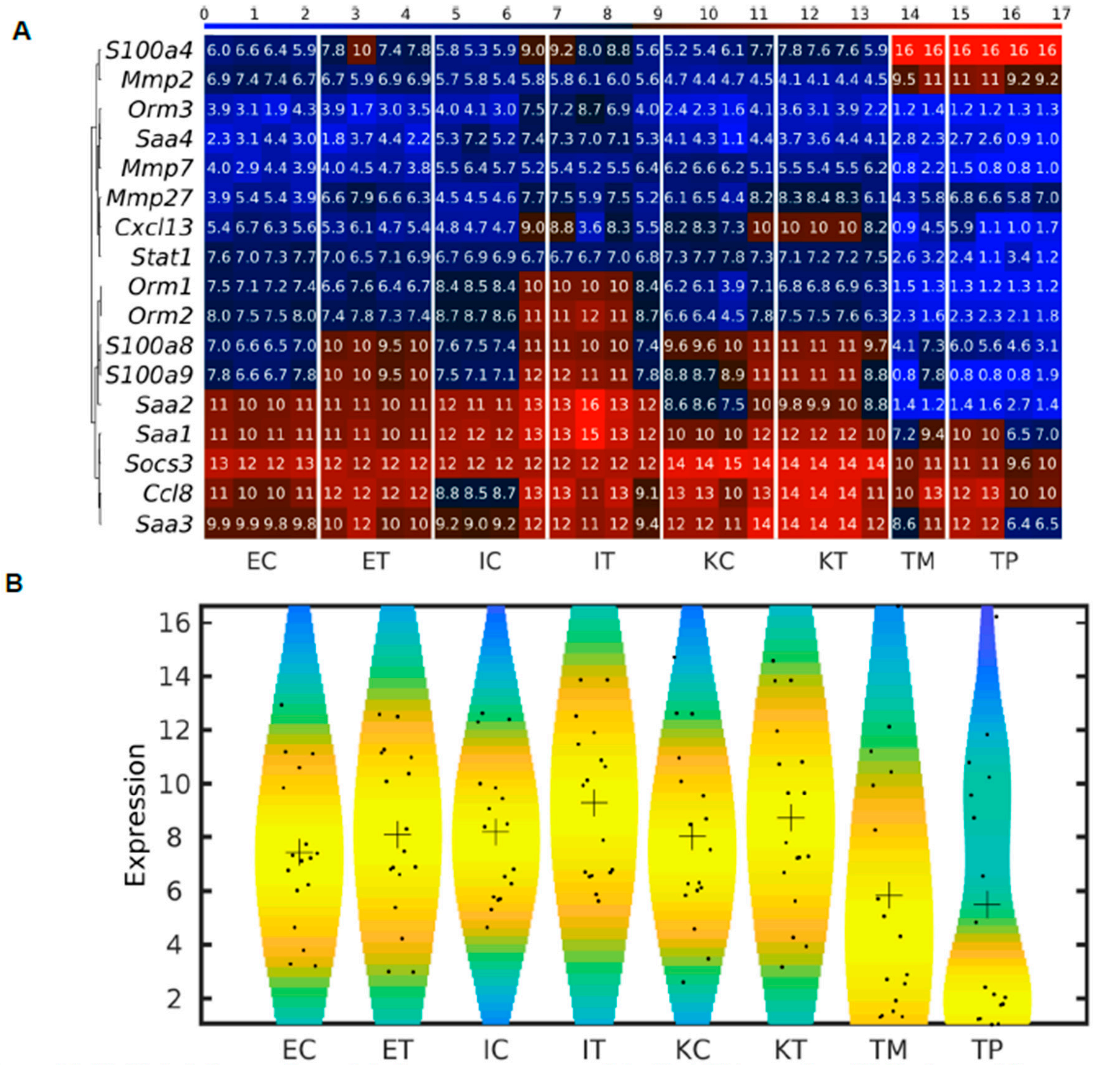

Figure S6. Multiple inflammation-related genes are up-regulated in TME samples. **(A)** Heatmap of the expression of inflammation related genes. Higher gene expression corresponds to redder color. (**B**) Violin plot of the expression distribution of inflammation related genes. The black crosses represent the position of the means. The black points represent the spread of the expression of the genes used to build the distributions. The samples are denoted with E, I and K for endothelial, Ito and Kupffer cells, and C and T, for control and TME cells, and TP and TM for CRC primary and tumor liver metastasis cells, respectively.

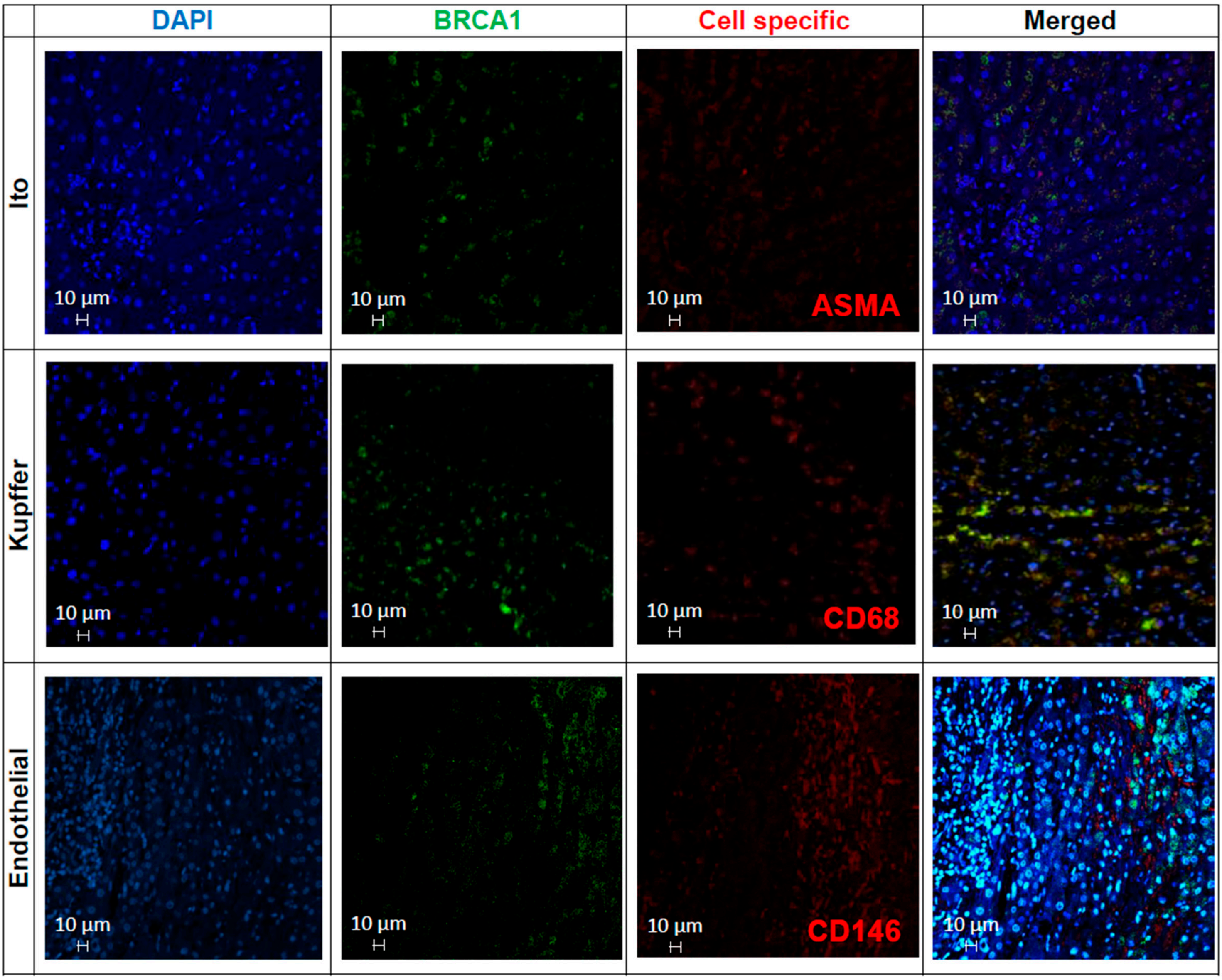

Figure S7. BRCA1 expression co-localization in TME cells from CRC liver metastases**.** BRCA1 co-localization on TMA samples on Ito, Kupffer and Endothelial cells from human CRC liver metastases. Magnified tumor surrounding area from Figure 7A shows a specific mark in each cell type.
